# Correction to “Long noncoding RNA SNHG3 interacts with microRNA‐502‐3p to mediate ITGA6 expression in liver hepatocellular carcinoma”

**DOI:** 10.1111/cas.70155

**Published:** 2025-07-18

Guo Guo J, Zhang J, Xiang Y, Zhou S, Yang Y, Zheng J. Long noncoding RNA SNHG3 interacts with microRNA‐502‐3p to mediate ITGA6 expression in liver hepatocellular carcinoma. *Cancer Sci*. 2024;115:2286‐2300. https://doi.org/10.1111/cas.16190.

In the above article, Dr. Jinfang Zheng's affiliation is incorrect. The correct affiliation is shown below:

Department of Hepatobiliary Surgery, Hainan General Hospital (Hainan Affiliated Hospital of Hainan Medical University), Haikou, Hainan, P.R. China.

We apologize for this error.

